# Inhibition of Breast Cancer Bone Metastasis by LRP5-Overexpressing Osteocytes via the LIMA1/MYO5B Signaling Axis

**DOI:** 10.3390/ijms27020777

**Published:** 2026-01-13

**Authors:** Yaning Chen, Zicheng Wang, Yu Sun, Xinshi Li, Yuji Wang, Shengzhi Liu

**Affiliations:** 1 Department of Pharmacology, College of Pharmaceutical Sciences of Capital Medical University, Beijing 100069, China; chenyaning@mail.ccmu.edu.cn (Y.C.);; 2 Beijing Area Major Laboratory of Peptide and Small Molecular Drugs, Engineering Research Center of Endogenous Prophylactic of Ministry of Education of China, Beijing Laboratory of Biomedical Materials, Beijing 100069, China; 3 Institute of Metallic Biomaterials, Helmholtz-Zentrum Hereon, 21502 Geesthacht, Germany; 4 Department of Medicinal Chemistry, College of Pharmaceutical Sciences of Capital Medical University, Beijing 100069, China; 5 Beijing Laboratory of Oral Health, Capital Medical University, Beijing 100069, China

**Keywords:** breast cancer, bone metastasis, osteocytes, conditioned medium, LRP5, LIMA1, MYO5B

## Abstract

Bone metastasis in breast cancer remains a major therapeutic challenge because current osteoclast-targeted therapies do not fully disrupt the tumor–bone vicious cycle. Osteocytes, the most abundant bone cells, are increasingly recognized as key regulators of bone–tumor crosstalk. Previous work has shown that osteocyte-specific overexpression of the Wnt co-receptor LRP5 inhibits breast cancer-induced osteolysis and generates conditioned medium (CM) with tumor-suppressive activity. Proteomic analysis identified LIM domain and actin-binding protein 1 (LIMA1) as a central mediator that interacts with Myosin Vb (MYO5B), suggesting the role of the LIMA1/MYO5B regulatory axis. This study demonstrates that CM derived from LRP5-overexpressing osteocytes suppresses EO771 breast cancer cell proliferation, migration, and invasion, and downregulates tumor-promoting proteins, including MMP9, Snail, IL-6, and TGF-β1, while upregulating the apoptosis-related protein cleaved caspase-3. These effects were largely reversed by knockdown of LIMA1 or MYO5B. In syngeneic mouse models of mammary tumors and bone metastasis, systemic administration of LRP5-overexpressing osteocyte-derived CM reduced tumor burden and osteolytic bone destruction, whereas genetic knockdown of LIMA1 in osteocytes or MYO5B in tumor cells abrogated these protective effects. Collectively, these findings indicate that LRP5 activation in osteocytes engages the LIMA1/MYO5B signaling axis that inhibits breast cancer progression and osteolysis, disrupts tumor–stromal interactions, and restores bone–tumor homeostasis, thereby providing a potential therapeutic strategy to break the vicious cycle of bone metastasis in breast cancer.

## 1. Introduction

Breast cancer is the second most commonly diagnosed cancer worldwide. Among women, it is the most frequently diagnosed malignancy, accounts for the highest cancer incidence, and remains the leading cause of cancer-related death [1,2]. Bone metastasis is a common and devastating complication, occurring in up to 75% of patients with advanced breast cancer [3,4]. Once established, bone metastases precipitate a spectrum of skeletal-related events, including pathological fractures, hypercalcemia, and intractable pain, which markedly compromise patient survival rates and quality of life [5]. Metastatic dissemination to bone involves tumor cell migration, invasion, and subsequent colonization of the bone marrow niche, where cancer cells secrete factors that stimulate osteoclast activation while suppressing osteoblast function. This disruption of the bone microenvironment ultimately drives the formation and progression of bone metastases [6,7]. Although agents targeting osteoclast activity, such as bisphosphonates and receptor activator of nuclear factor-κ B ligand (RANKL) inhibitors, have improved clinical management, they do not fully interrupt the “vicious cycle” in which tumor-induced bone destruction and bone microenvironment-driven tumor progression reinforce one another, underscoring an urgent need for novel strategies [8]. Mounting evidence highlights the dual role of osteocytes in maintaining bone homeostasis and modulating tumor behavior. As the most abundant and long-lived cells in bone, osteocytes play crucial functions in regulating skeletal health and are increasingly recognized as key regulators of bone–tumor interactions and skeletal health [9,10,11].

Previous studies have shown that upregulation of low-density lipoprotein receptor-related protein 5 (LRP5) in osteocytes exerts inhibitory effects on tumor cells [12,13]. Although Wnt signaling is frequently associated with tumor-promoting functions, LRP5, as a co-receptor in the canonical Wnt pathway, is essential for bone protection, formation, and mechanotransduction [14]. In osteocytes, LRP5 not only mediates load-induced bone anabolism but also inhibits tumor-driven osteolysis in breast cancer models. Osteocyte-specific LRP5 overexpression endows osteocytes with tumor-suppressive properties, resulting in the secretion of conditioned medium (CM) enriched in factors that inhibit breast cancer cell proliferation and metastasis [15,16].

Our proteomic analysis identified LIM domain and actin-binding protein 1 (LIMA1) as a key mediator in this process, with its expression markedly upregulated in CM derived from LRP5-overexpressing osteocytes [12]. LIMA1 is a regulator of actin cytoskeleton dynamics and epithelial–mesenchymal transition (EMT) and has been proposed as a potential metastasis suppressor [17,18]. Downregulation of LIMA1 is associated with tumor progression in breast, prostate, and oral cancers, whereas restoration of its expression inhibits cancer cell migration and invasion [19,20]. Notably, mass spectrometry-based proteomics identified myosin Vb (MYO5B) as a major LIMA1-interacting protein. MYO5B is a motor protein essential for vesicle trafficking and cholesterol uptake, and its dysregulation perturbs intracellular transport and cytoskeletal organization [21]. These observations led us to hypothesize that LRP5-overexpressing osteocytes inhibit breast cancer bone metastasis by engaging the LIMA1/MYO5B signaling axis, thereby disrupting tumor–stromal interactions and restoring bone–tumor homeostasis [22].

We therefore investigated the effects of LRP5-overexpressing osteocyte-derived CM on the expression of proteins associated with tumor progression (e.g., MMP9, Snail, cleaved caspase-3, IL-6, TGF-β1) and on breast cancer cell proliferation, migration, and invasion of tumor cells [23]. The contribution of the LIMA1/MYO5B axis was further validated by selectively modulating LIMA1 and MYO5B gene expression. Beyond characterizing the inhibition of tumor progression, we also examined the regulatory effects of LRP5-overexpressing osteocyte-derived CM on RANKL-induced osteoclast differentiation and immune cell activation. In mouse models of breast cancer and breast cancer bone metastasis, LRP5-overexpressing osteocyte-derived CM markedly reduced tumor burden and osteolytic lesions, whereas genetic knockdown of either LIMA1 or MYO5B abolished these protective effects. Together, our results demonstrate that LRP5 in osteocytes inhibits effects on breast cancer cells primarily by activating the LIMA1/MYO5B signaling axis. This work highlights the pivotal role of osteocytes-the most abundant and highly plastic bone cells—as active regulators that shape the tumor microenvironment. LRP5 appears to convert a tumor-driven vicious cycle into a bone-protective positive feedback loop, underscoring the therapeutic potential of targeting osteocytes in breast cancer and its bone metastases. By elucidating the LIMA1/MYO5B signaling axis, we provide a conceptual framework for osteocyte-based therapy and identify potential molecular targets for restoring bone–tumor homeostasis. Collectively, our findings define the LRP5/LIMA1/MYO5B axis as a novel signaling pathway that repurposes a canonical Wnt signaling component for tumor suppression.

## 2. Results

### 2.1. LRP5-Overexpressing Osteocyte-Derived CM Inhibits EO771 Cell Proliferation, Migration, and Invasion

LRP5 in osteocytes plays a critical role in tumor suppression. Building on our previous observation that conditioned medium from LRP5-overexpressing osteocytes inhibits breast cancer cells, we sought to further validate the anti-tumor capacity of osteocytes [12]. To this end, MLO-A5 osteocytes were transfected with an LRP5-overexpressing plasmid, and CM was collected from these cells. EO771 breast cancer cells were then cultured in this CM, and their proliferation, migration, and invasion were evaluated (Supplementary Figure S1B–E). LRP5-overexpressing osteocyte-derived CM significantly reduced EO771 cell viability, indicating suppressed proliferative activity, and markedly impaired their migration and invasion capacities. Western blot analysis of key proteins involved in metastasis, apoptosis, and inflammation revealed that treatment with LRP5-overexpressing osteocyte-derived CM downregulated tumor-promoting proteins, including MMP9, Snail, TGF-β1, and IL-6, while upregulating the apoptosis-related protein cleaved caspase-3 (Supplementary Figure S1F). These findings demonstrate that LRP5 overexpression confers anti-tumor properties on osteocytes and that factors secreted into their CM can inhibit tumor cell proliferation, migration, and invasion. Consistently, treatment of 4T1.2 and MCF-7 cells with LRP5-overexpressing osteocyte-derived CM also produced robust anti-tumor effects, further validating our previous observations (Supplementary Figure S2).

### 2.2. Role of LIMA1 in the Anti-Tumor Effects of LRP5-Overexpressing Osteocyte-Derived CM

**Figure 1:**
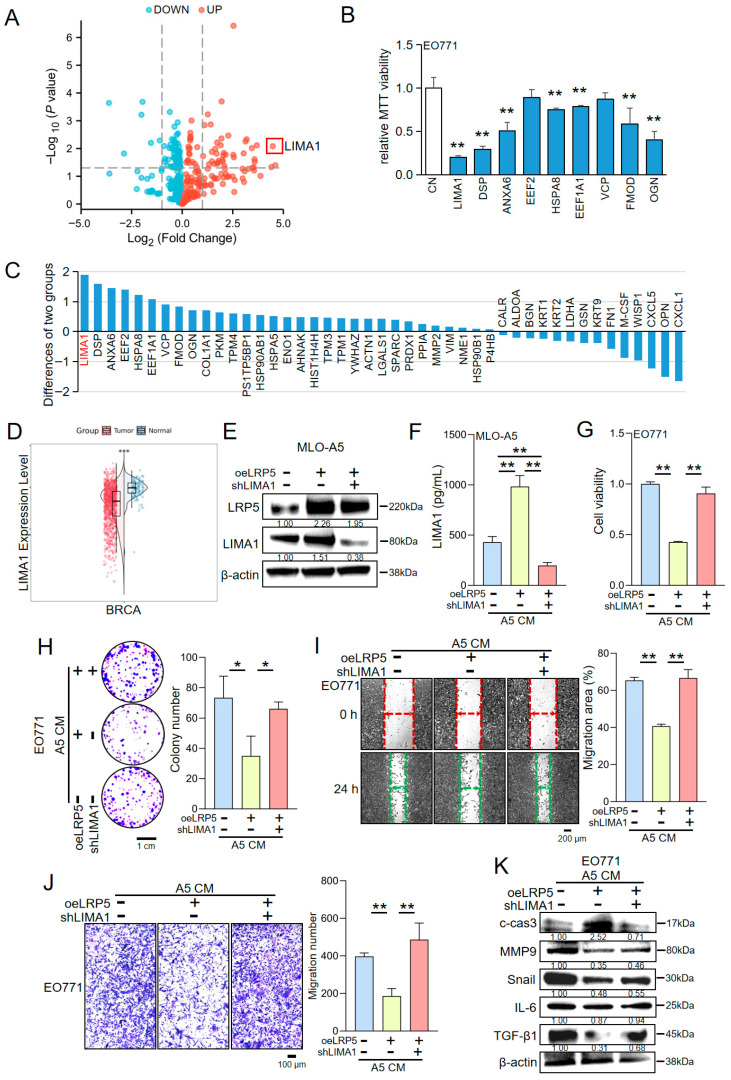
LIMA1 mediates the tumor-suppressive effects of conditioned medium (CM) derived from LRP5-overexpressing osteocytes. CN = Control, CM = Conditioned medium, oeLRP5 = overexpressed LRP5, shLIMA1 = LIMA1 shRNA. The single, double, and triple asterisks indicate *p* < 0.05, *p* < 0.01, and *p* < 0.001, respectively. (**A**) Quantitative mass spectrometry-based proteomic analysis of conditioned media from Lrp5-overexpressing MLO-A5 osteocytes versus control cells. Significantly upregulated (red) and downregulated (blue) genes were defined by an adjusted *p*-value < 0.05 and |Log_2_FC| > 1 (dashed lines). (**B**) Selected recombinant proteins corresponding to the most significantly upregulated candidates were applied to the mouse EO771 mouse breast cancer cells, and their effects on cell proliferation were assessed by MTT assay. (**C**) Comparative analysis of proteins secreted by Lrp5-overexpressing osteocytes versus control osteocytes, showing upregulated (positive values) and downregulated (negative values) proteins. (**D**) LIMA1 expression in normal tissues and in breast cancer patients. (**E**) Modulation of LIMA1 levels in MLO-A5 osteocytes following LRP5 overexpression and/or LIMA1 knockdown. (**F**) ELISA quantification of LIMA1 in the LRP5-overexpressing osteocyte-derived CM before and after LIMA1 knockdown. (**G**,**H**) Effects of LIMA1 knockdown on the ability of LRP5-overexpressing osteocyte-derived CM to inhibit EO771 cell proliferation. (**I**,**J**) Effects of LIMA1 knockdown on the inhibitory action of LRP5-overexpressing osteocyte-derived CM on EO771 cell migration and invasion. (**K**) Expression of metastasis- and apoptosis-related proteins in EO771 cells treated with LRP5-overexpressing osteocyte-derived CM after LIMA1 knockdown. Samples were derived from parallel experiments.

To identify the specific mediators underlying the anti-tumor activity of LRP5-overexpressing osteocyte-derived CM, we performed a mass spectrometry-based proteomic analysis. This revealed a distinct secretory profile, with multiple proteins being upregulated in CM from LRP5-overexpressing osteocytes compared with control osteocytes (Figure 1A). Gene Ontology (GO) and Kyoto Encyclopedia of Genes and Genomes (KEGG) enrichment analyses indicated that these secreted proteins were predominantly associated with pathways regulating cell motility and adhesion (Supplementary Figure S1G). Concurrently, GSEA validated the activation of cytoskeleton and motility pathways (Supplementary Figure S5A–E). Among these, LIMA1 was one of the most markedly elevated proteins (Figure 1C). EO771 cells were treated with recombinant proteins corresponding to the top upregulated hits identified in the screen, and cell proliferation was assessed using an MTT assay (Figure 1B). Among these proteins, one exerted the strongest anti-proliferative effect. Consistently, analysis of The Cancer Genome Atlas (TCGA) database showed that LIMA1 transcript levels were significantly lower in patients with invasive breast carcinoma than in healthy controls (Figure 1D) [24]. Meanwhile, we also conducted a comparative analysis of overall survival (OS) and recurrence-free survival (RFS) using the Kaplan–Meier survival plot [25]. The results indicate that patients with high LIMA1 expression have a higher overall survival rate than those with low LIMA1 expression (Supplementary Figure S1H). To elucidate how LIMA1 mediates the tumor-suppressive effects of LRP5-overexpressing osteocyte-derived CM, we generated an MLO-A5 osteocyte model with LIMA1 knockdown combined with LRP5 overexpression using plasmid and shRNA transfection. Western blot and ELISA analyses demonstrated that LRP5 overexpression increased LIMA1 expression both in osteocytes and in their CM, whereas this upregulation was attenuated in cells in which LIMA1 was knocked down (Figure 1E,F). CCK-8 proliferation assays, colony formation assays, two-dimensional motility assays, and transwell assays showed that LIMA1 knockdown diminished the inhibitory effects of LRP5-overexpressing osteocyte-derived CM on EO771 cell proliferation, migration, and invasion (Figure 1G–J). Western blot analysis further revealed that LIMA1 knockdown reversed the regulatory effects of LRP5-overexpressing osteocyte-derived CM on tumor-associated proteins: the metastasis-related markers Snail, MMP9, IL-6, and TGF-β1 were upregulated, whereas the expression of the apoptosis-related protein cleaved caspase-3 was reduced (Figure 1K). Collectively, these findings indicate that LIMA1 functions as a downstream effector of the LRP5/Wnt signaling axis in mediating the anti-tumor actions of LRP5-overexpressing osteocyte-derived CM, and that its knockdown substantially attenuates these inhibitory effects.

### 2.3. Suppression of EO771 Cells by Recombinant LIMA1 Protein

**Figure 2:**
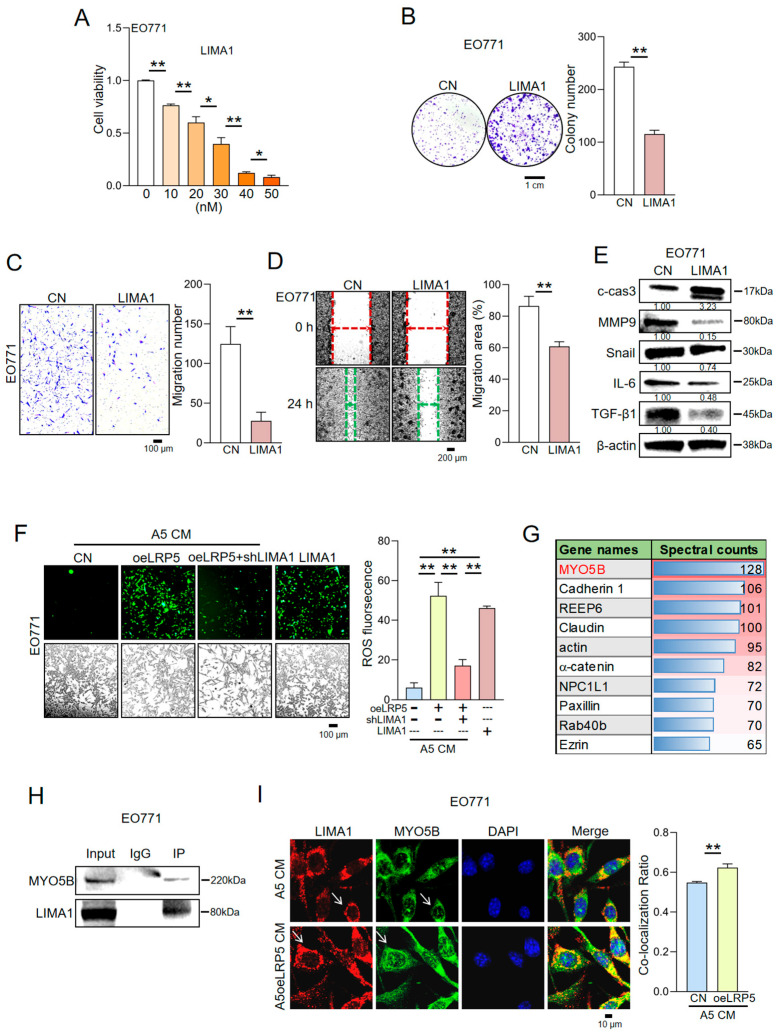
Inhibitory effect of recombinant LIMA1 protein on breast cancer cells. CN = Control, CM = Conditioned medium, oeLRP5 = overexpressed LRP5, shLIMA1 = LIMA1 shRNA, ROS = Reactive oxygen species. The single and double asterisks indicate *p* < 0.05 and *p* < 0.01, respectively. (**A**,**B**) Inhibitory effects of recombinant LIMA1 protein on EO771 cell proliferation. (**C**,**D**) Inhibitory effects of recombinant LIMA1 protein on EO771 cell migration and invasion. (**E**) Protein expression levels in EO771 cells in response to LIMA1 treatment. Samples were derived from parallel experiments. (**F**) LIMA1 regulates ROS production in EO771 cells. (**G**) Proteins co-immunoprecipitated with LIMA1 were subjected to mass spectrometric analysis, and MYO5B was identified as a key binding partner of LIMA1. MYO5B was identified as a key binding partner of LIMA1. (**H**) Co-immunoprecipitation of LIMA1 with MYO5B. (**I**) Co-localization of LIMA1 and MYO5B in EO771 cells as detected by immunofluorescence. The white arrows point to the LIMA1 and MYO5B proteins.

To directly validate the contribution of LIMA1, EO771 cells were treated with recombinant LIMA1 protein. Exogenous LIMA1 significantly inhibited EO771 cell proliferation, as determined by CCK-8 and colony formation assays, and reduced cell motility and invasion in two-dimensional motility and transwell assays, respectively, producing effects highly consistent with those of LRP5-overexpressing osteocyte-derived CM (Figure 2A–D). Western blot analysis confirmed that recombinant LIMA1 recapitulated the regulatory effects of LRP5-overexpressing osteocyte-derived CM on proteins associated with metastasis and pro-inflammatory signaling (Figure 2E). Furthermore, reactive oxygen species (ROS) detection assays showed that both LRP5-overexpressing osteocyte-derived CM and recombinant LIMA1 induced ROS production in EO771 cells, whereas this effect was attenuated when LIMA1 was knocked down (Figure 2F). Together, these results confirm that LIMA1 is a major contributor to the anti-tumor activity of CM derived from LRP5-overexpressing osteocytes.

### 2.4. The LIMA1-MYO5B Signaling Axis Mediates the Anti-Tumor Effects of LRP5-Overexpressing Osteocyte-Derived CM

To investigate the mechanism by which LIMA1 inhibits tumor progression, we isolated LIMA1-binding proteins by co-immunoprecipitation and analyzed them using mass spectrometry. This proteomic approach identified MYO5B as a putative LIMA1-interacting partner (Figure 2G). We further validated this interaction by performing co-immunoprecipitation with protein lysates from EO771 cells, followed by Western blotting, which confirmed a physical association between LIMA1 and MYO5B (Figure 2H). Immunofluorescence staining combined with confocal microscopy demonstrated co-localization of LIMA1 and MYO5B in EO771 cells (Figure 2I). This suggests that LIMA1 can enter tumor cells through a specific mechanism, where it binds to MYO5B and co-localizes with it. Together, these findings indicate that LIMA1 exerts its anti-tumor effects, at least in part, through interaction with MYO5B in EO771 cells.

### 2.5. Role of MYO5B in the LIMA1-MYO5B Signaling Axis

**Figure 3:**
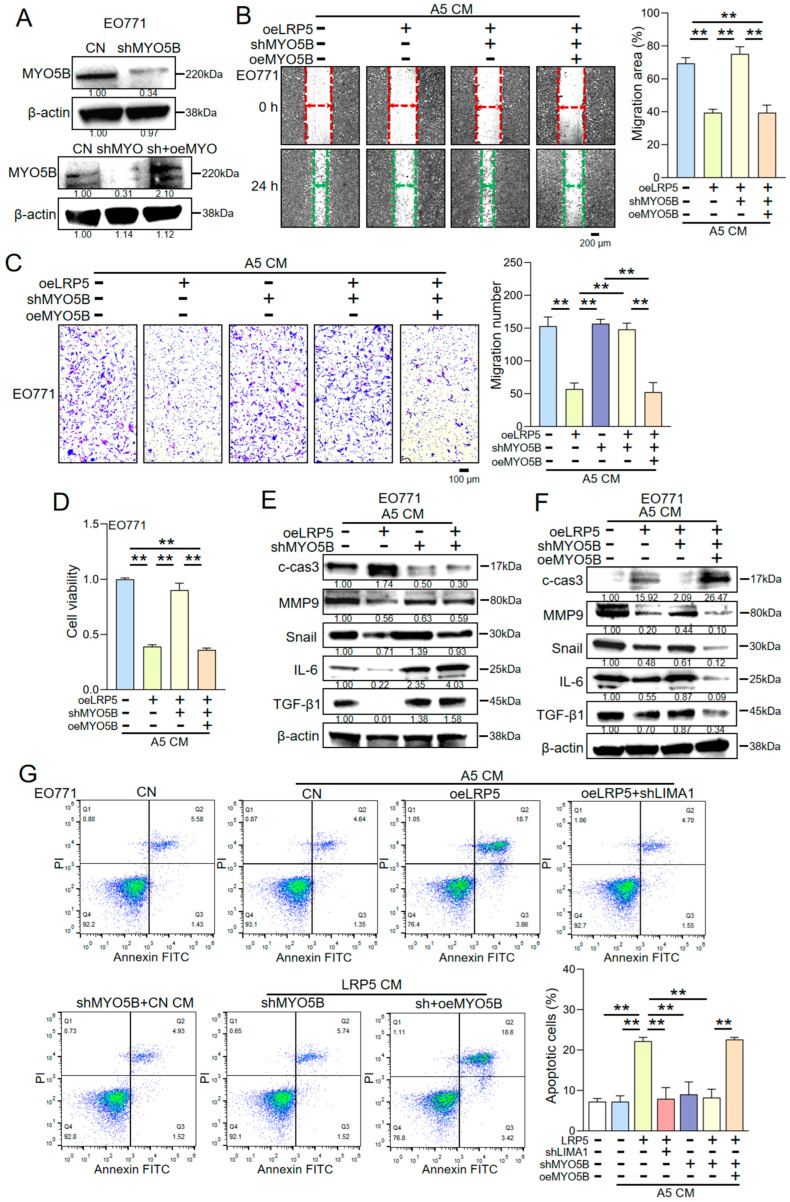
Role of MYO5B in the tumor-suppressive capacity of LRP5-overexpressing osteocyte-derived CM. CN = Control, CM = Conditioned medium, oeLRP5 = overexpressed LRP5, shLIMA1 = LIMA1 shRNA, shMYO5B = MYO5B shRNA, oeMYO5B = overexpressed MYO5B. The double asterisk indicates *p* < 0.01. (**A**) CCK-8 assay showing the effect of MYO5B knockdown on EO771 cell proliferation in response to LRP5-overexpressing osteocyte-derived CM. (**B**) Colony formation assay and quantitative analysis of MYO5B knockdown effects. (**C**) Two-dimensional motility assay assessing the effect of MYO5B knockdown on EO771 cell migration in response to LRP5-overexpressing osteocyte-derived CM. (**D**) Transwell invasion assay and quantification of EO771 cell invasion following treatment with LRP5-overexpressing osteocyte-derived CM. (**E**) Western blot analysis showing that MYO5B knockdown reverses LRP5-overexpressing osteocyte-derived CM-mediated regulation of metastasis-related and apoptosis-related proteins in EO771 cells. (**F**) Alterations in protein expression induced by LRP5-overexpressing osteocyte-derived CM in EO771 cells with MYO5B reconstitution. Samples were derived from parallel experiments. (**G**) Flow cytometry analysis of apoptosis induction in EO771 cells by LRP5-overexpressing osteocyte-derived CM, and the roles of LIMA1 and MYO5B.

**Figure 4:**
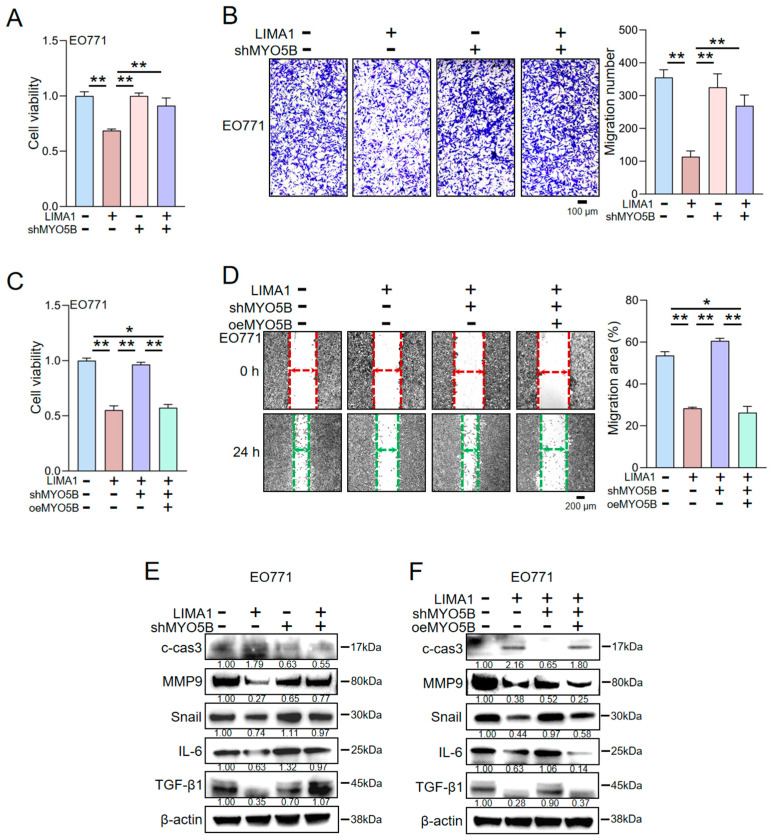
Effect of MYO5B on the tumor-suppressive capacity of recombinant LIMA1 protein. CN = Control, CM = Conditioned medium, shMYO5B = MYO5B shRNA, oeMYO5B = overexpressed MYO5B. The single and double asterisks indicate *p* < 0.05 and *p* < 0.01, respectively. (**A**,**B**) Dependence of LIMA1-mediated suppression of EO771 cell proliferation and invasion on MYO5B expression. (**C**,**D**) Restoration of LIMA1-mediated suppression of EO771 cell proliferation and migration upon MYO5B reconstitution. (**E**) Western blot analysis of metastasis- and apoptosis-related proteins in EO771 cells treated with recombinant LIMA1, with or without MYO5B knockdown. (**F**) Schematic illustration of the proposed mechanism by which MYO5B mediates the tumor-suppressive effects of LIMA1 in EO771 cells. Samples were derived from parallel experiments.

The functional contribution of MYO5B to this axis was examined by knocking down MYO5B expression in EO771 cells using shRNA (Figure 3A). MYO5B silencing attenuated the inhibitory effects of LRP5-overexpressing osteocyte-derived CM on EO771 cell proliferation, as demonstrated by CCK-8 and colony formation assays (Supplementary Figure S3A,B). Moreover, MYO5B knockdown counteracted the LIMA1-mediated downregulation of MMP9, Snail, IL-6, and TGF-β1 and blunted the increase in cleaved caspase-3 induced by LRP5-overexpressing osteocyte-derived CM (Figure 3E). Consistently, the suppressive effects of LRP5-overexpressing osteocyte-derived CM on EO771 cell migration and invasion were largely abolished upon MYO5B knockdown (Figure 3C; Supplementary Figure S3D). These results indicate that the anti-tumor activity of LRP5-overexpressing osteocyte-derived CM is mediated, at least in part, through the LIMA1-MYO5B signaling axis in EO771 cells. To further validate the role of MYO5B, we performed rescue experiments by re-expressing MYO5B in EO771 cells in which endogenous MYO5B had been silenced. Reintroduction of MYO5B via an overexpression plasmid restored the anti-proliferative effects of LRP5-overexpressing osteocyte-derived CM in CCK-8 and colony formation assays (Figure 3D, Supplementary Figure S3C). Western blot analysis confirmed that the expression levels of MMP9, Snail, IL-6, TGF-β1, and cleaved caspase-3 returned to levels comparable to those observed in control cells treated with LRP5-overexpressing osteocyte-derived CM (Figure 3F). Flow cytometry further showed that LRP5-overexpressing osteocyte-derived CM increased the proportion of apoptotic EO771 cells compared with control CM, whereas MYO5B knockdown reduced this pro-apoptotic effect. MYO5B re-expression again rescued the diminished apoptosis (Figure 3G) and reinstated the inhibitory effects of LRP5-overexpressing osteocyte-derived CM on EO771 cell migration and invasion (Figure 3B). Collectively, these findings demonstrate that LRP5-overexpressing osteocyte-derived CM exerts its anti-tumor effects on EO771 cells primarily through the LIMA1–MYO5B signaling axis, with LIMA1 acting functionally via MYO5B. To investigate MYO5B’s necessity for LIMA1’s tumor-suppressive function, MYO5B-knockdown EO771 cells were treated with recombinant LIMA1 protein. Notably, LIMA1’s anti-tumor activity was significantly attenuated in these cells. The inhibitory effects of LIMA1 on cell proliferation, migration, and invasion were diminished (Figure 4A,B; Supplementary Figure S4A,B). Following MYO5B reconstitution, the suppressive effects of LIMA1 on proliferation, migration, and invasion were restored (Figure 4C,D; Supplementary Figure S4A,C). Furthermore, MYO5B influenced LIMA1-induced protein expression changes consistently with LRP5-overexpressing osteocyte-derived CM (Figure 4E,F). Collectively, these results demonstrate that LIMA1 exerts its inhibitory effect on EO771 cells through a functional interaction with MYO5B.

### 2.6. Regulatory Effects of LIMA1 on Osteoclasts and Vascular Endothelial Cells

**Figure 5:**
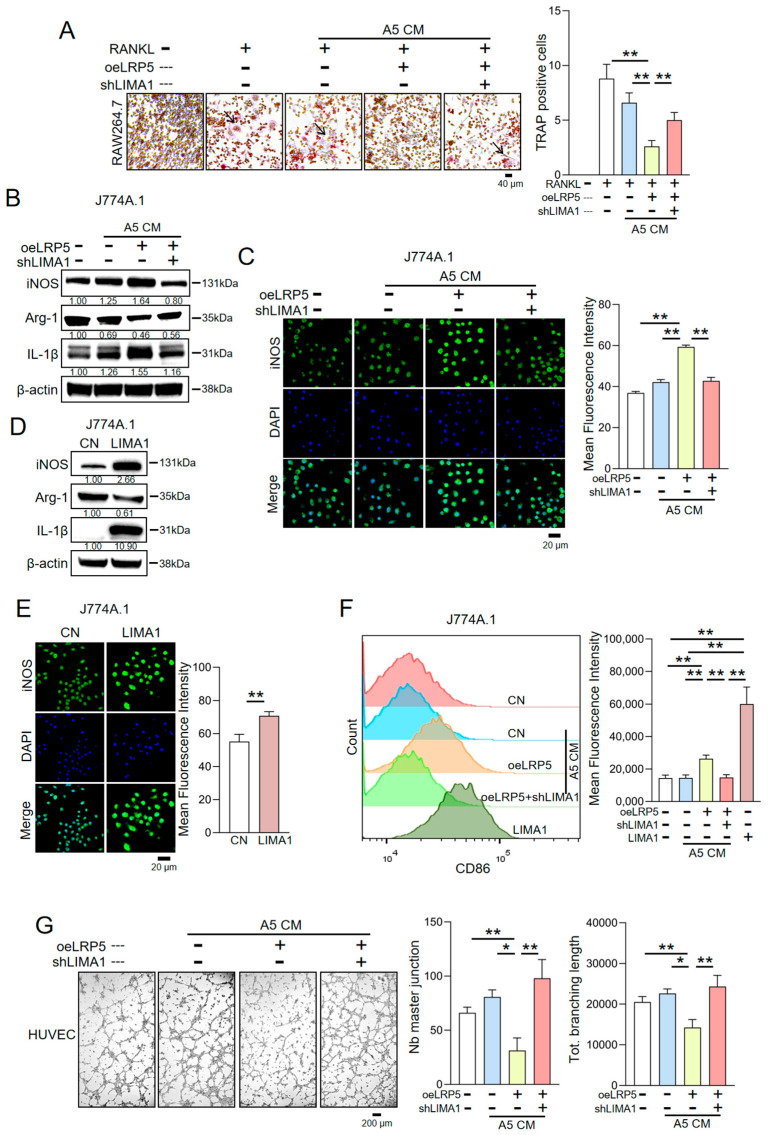
Effect of LIMA1 in LRP5-overexpressing osteocyte-derived CM on osteoclasts, immune cells, and vascular endothelial cells. CN = Control, CM = Conditioned medium, oeLRP5 = overexpressed LRP5, shLIMA1 = LIMA1 shRNA. The single and double asterisks indicate *p* < 0.05 and *p* < 0.01, respectively. (**A**) TRAP staining and quantitative analysis showing that LRP5-overexpressing osteocyte-derived CM inhibits RANKL-induced osteoclast differentiation in RAW264.7 cells, whereas CM from LIMA1-knockdown osteocytes exhibits reduced inhibitory effects. The black arrows indicate TRAP-positive multinucleated cells. (**B**,**C**) LIMA1-dependent promotion of M1 polarization in J774A.1 macrophage in response to LRP5-overexpressing osteocyte-derived CM. (**D**,**E**) M1 polarization and protein expression changes in J774A.1 cells induced by the LIMA1 protein. Samples were derived from parallel experiments. (**F**) CD86 expression in J774A.1 cells after LIMA1 stimulation, measured by flow cytometry. (**G**) Inhibitory effect of LRP5-overexpressing osteocyte-derived CM on the tube-forming ability of HUVECs.

Osteoclasts play a pivotal role in bone resorption during breast cancer bone metastasis. To determine whether LIMA1 participates in the regulation of osteoclast differentiation in the context of LRP5-overexpressing osteocyte-derived CM, we used RAW264.7 cells as osteoclast precursors and treated them with RANKL under different CM conditions. Tartrate-resistant acid phosphatase (TRAP) staining and quantitative analysis revealed that LRP5-overexpressing osteocyte-derived CM significantly inhibited RANKL-induced osteoclast formation, as evidenced by a reduced number of TRAP-positive multinucleated cells. In contrast, CM derived from LIMA1-knockdown osteocytes exhibited a markedly diminished ability to inhibit osteoclast differentiation (Figure 5A). These findings indicate that LIMA1 within LRP5-overexpressing osteocyte-derived CM contributes to the suppression of osteoclast differentiation and thereby exerts a bone-protective effect.

In parallel, we utilized HUVECs to assess the impact of conditioned media from osteocytes overexpressing LRP5 on the process of angiogenesis. (Figure 5G). Results showed that CM collected from EO771 cells pre-treated with LRP5-overexpressing osteocyte-derived CM impaired the tube-forming capacity of HUVECs. In contrast, CM from EO771 cells pre-treated with LIMA1-knockdown LRP5-overexpressing osteocyte-derived CM restored HUVEC tube formation. This suggests that LRP5-overexpressing osteocyte-derived CM, via LIMA1, can modulate the pro-angiogenic capacity of EO771 cells, thereby inhibiting HUVEC-mediated angiogenesis.

### 2.7. LIMA1 Induces Immunoactivation of Macrophage J774A.1 Cells

To investigate the immunomodulatory role of LIMA1 in the context of LRP5-overexpressing osteocyte-derived CM, we used the murine macrophage cell line J774A.1 and assessed M1/M2 polarization in response to CM treatment. LRP5-overexpressing osteocyte-derived CM upregulated the expression of iNOS, IL-1β, and CD86 markers associated with M1 polarization—while downregulating Arg1 and CD206, which are characteristic of M2 macrophages (Figure 5B,C). Flow cytometry confirmed an increased proportion of CD86-positive cells, indicative of enhanced M1 polarization (Figure 5D,E). In contrast, CM from LIMA1-knockdown osteocytes failed to induce these M1 marker changes in J774A.1 cells. Direct treatment of J774A.1 cells with recombinant LIMA1 recapitulated the effects of LRP5-overexpressing osteocyte-derived CM on M1/M2 marker expression. Together, these findings suggest that LIMA1 in LRP5-overexpressing osteocyte-derived CM promotes an anti-tumor immune phenotype by driving M1 macrophage polarization in an LIMA1-dependent manner.

### 2.8. LRP5-Overexpressing Osteocyte-Derived CM Suppresses Tumor Growth and Protects Bone In Vivo

**Figure 6:**
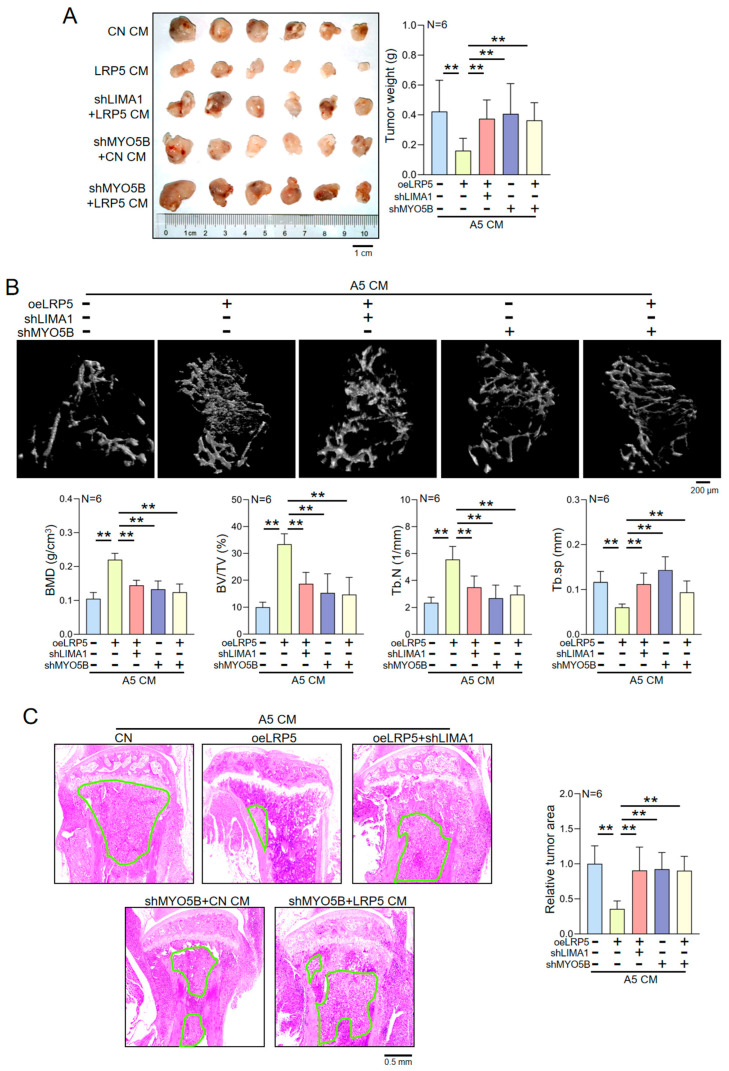
Inhibitory effect of LRP5-overexpressing osteocyte-derived CM on mammary tumors and tibial bone metastasis in C57BL/6 mice. CN = Control, CM = Conditioned medium, oeLRP5 = overexpressed LRP5, shMYO5B = MYO5B shRNA, shLIMA1 = LIMA1 shRNA. The double asterisk indicates *p* < 0.01. (**A**) Effects of control CM and LRP5-overexpressing osteocyte-derived CM on mammary tumor growth (tumor volume and weight) in C57BL/6 mice (N = 6). (**B**) LRP5-overexpressing osteocyte-derived CM reduces bone loss at tumor-invaded tibial sites, as shown by micro-CT reconstruction and quantitative analysis of bone parameters (N = 6). BV/TV = Bone Volume/Tissue Volume, BMD = Bone Mineral Density, Tb.N = Trabecular Number, Tb.Sp = Trabecular Separation. (**C**) LRP5-overexpressing osteocyte-derived CM significantly inhibited EO771 breast cancer cell invasion into the tibia (highlighted by green boxes) (N = 6).

To validate the tumor-suppressive and bone-protective effects of LRP5-overexpressing osteocyte-derived CM in vivo, we established syngeneic mammary tumor and tibial bone metastasis models in C57BL/6 mice. EO771 cells were injected into the mammary fat pad or the tibial bone marrow cavity, followed by systemic administration of control CM or LRP5-overexpressing osteocyte-derived CM via intraperitoneal injection. In the mammary tumor model, treatment with LRP5-overexpressing osteocyte-derived CM significantly reduced tumor volume and weight compared with the control CM (Figure 6A). In the tibial bone metastasis model, micro-computed tomography (micro-CT) analysis showed that LRP5-overexpressing osteocyte-derived CM increased trabecular bone volume and preserved cortical bone integrity at sites of tumor invasion, thereby attenuating bone loss (Figure 6B). In contrast, LRP5-overexpressing CM derived from LIMA1-knockdown osteocytes failed to show significant inhibition of tumor progression or protection against bone destruction in the above mouse models. Moreover, in mice inoculated with MYO5B-knockdown EO771 cells, the therapeutic effect of LRP5-overexpressing osteocyte-derived CM was markedly reduced. Compared with normal EO771 tumors treated with LRP5-overexpressing osteocyte-derived CM, tumors formed by MYO5B-knockdown EO771 cells were larger and caused more severe osteolytic damage, even in the presence of LRP5-overexpressing CM. These results indicate that the absence of MYO5B in tumor cells diminishes the therapeutic efficacy of LRP5-overexpressing osteocyte-derived CM. Histological analysis of H&E-stained tibial sections further revealed that the tumor infiltration area was significantly smaller in mice treated with LRP5-overexpressing osteocyte-derived CM than in the control group (Figure 6C). The in vivo experiments also support a role for the LIMA1-MYO5B axis in this process: mice receiving LRP5-overexpressing CM from LIMA1-knockdown osteocytes exhibited greater tumor infiltration than those treated with normal LRP5-overexpressing CM, whereas the tumor-suppressive effect of LRP5-overexpressing CM was significantly attenuated in mice bearing MYO5B-knockdown EO771 tumors. In summary, our data demonstrate that LRP5-overexpressing osteocyte-derived CM exerts anti-tumor and bone-protective effects in vivo, and that LIMA1 and MYO5B may be relevant factors contributing to these actions within the bone microenvironment.

**Figure 7:**
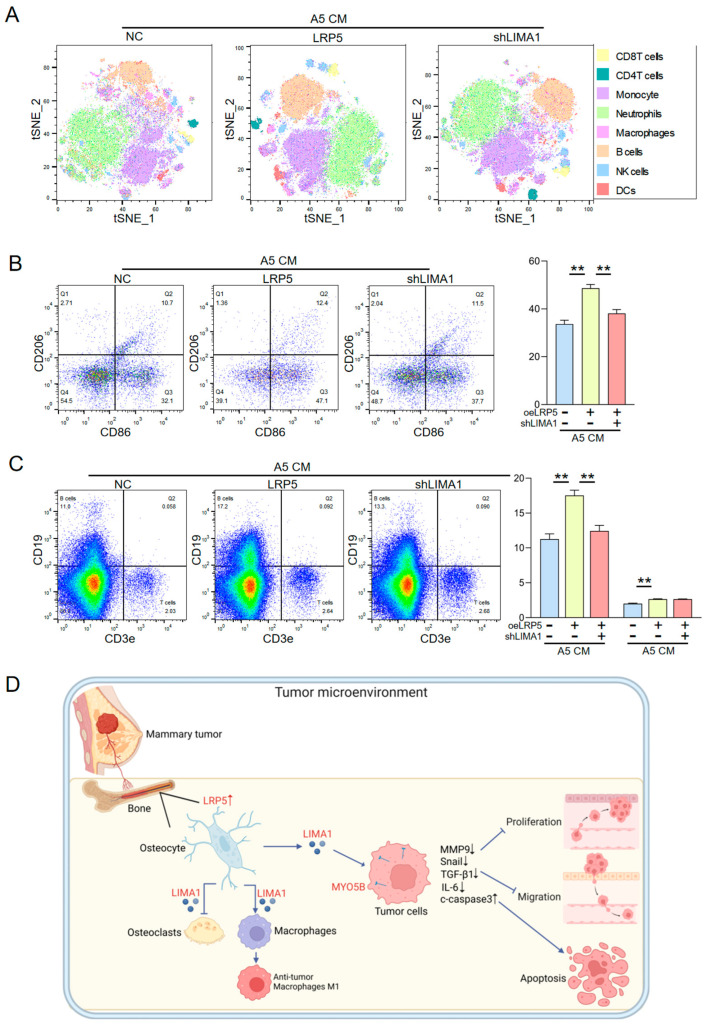
Immune cell populations and the regulatory mechanism of LRP5-overexpressing osteocyte-derived CM. CN = Control, CM = Conditioned medium, oeLRP5 = overexpressed LRP5, shLIMA1 = LIMA1 shRNA. The double asterisk indicates *p* < 0.01. (**A**) Single-cell mass cytometry t-SNE plots showing immune cell populations in bone marrow from tumor-bearing mice treated with control CM or LRP5-overexpressing osteocyte-derived CM. (**B**) Characterization of macrophage populations. (**C**) Profiling of B and T lymphocyte populations. (**D**) Proposed mechanism by which LRP5-overexpressing osteocyte-derived CM regulates tumor cells and the immune microenvironment. The interaction between MYO5B, a cytoplasmic motor protein, and LIMA1 is likely to occur within the cytoplasmic compartment or during vesicular transport processes.

To assess changes in immune cell heterogeneity, we performed single-cell mass cytometry in a mouse model of breast cancer bone metastasis and visualized the data using t-SNE (Figure 7A). The analysis revealed that treatment with LRP5-overexpressing osteocyte-derived CM was associated with an anti-tumor immune profile in the bone marrow, including increased proportions of NK cells, B cells, and T cells. Further immune profiling indicated that this CM promoted a shift in macrophage polarization toward the M1 phenotype, which is generally associated with tumor-killing activity (Figure 7B). This immunostimulatory pattern was markedly reduced when LIMA1 was knocked down, suggesting that LIMA1 may contribute to the pro-inflammatory, anti-tumor immune microenvironment induced by LRP5-overexpressing osteocyte-derived CM. Consistent with these observations, elevated proportions of B and T cells were also detected after treatment with LRP5-overexpressing osteocyte-derived CM (Figure 7C). Together, these data indicate that LIMA1 is involved in mediating the observed immune changes, particularly in the promotion of M1-like macrophage polarization.

## 3. Discussion

This study elucidates how the LRP5/Wnt signaling axis mediates anti-tumor effects by regulating the secretion of LIMA1 from osteocytes. LRP5, a co-receptor for Wnt ligands, is indispensable for Wnt signal transduction and contributes to cancer progression through pathways such as Wnt/β-catenin [26,27]. LIMA1, a central regulator of actin cytoskeleton homeostasis, controls cytoskeletal dynamics, cell morphology, migration, and adhesion, and is known to maintain osteoblast morphology and focal adhesion maturation. Previous studies have reported that LIMA1 expression is downregulated in several epithelial cancers, including breast and prostate cancer [20]. MYO5B, a member of the myosin family, facilitates cytoskeletal remodeling and intracellular trafficking, and its dysregulation has been linked to enhanced tumor cell migration and invasion. Once breast cancer cells metastasize to bone, they engage in reciprocal crosstalk with osteolineage cells. In response, osteoblasts and osteocytes can release anti-tumor factors to counteract tumor invasion (Figure 7D). Here, we show that conditioned medium from LRP5-overexpressing MLO-A5 osteocytes markedly inhibits EO771 breast cancer cell proliferation, migration, and invasion, and alters the expression of key tumor-related proteins—effects that are mediated by upregulation of LIMA1. LIMA1 functions, at least in part, through interaction with MYO5B within tumor cells, thereby activating downstream signaling that suppresses the expression of metastasis-associated proteins (MMP9, Snail, TGF-β1) and the pro-inflammatory cytokine IL-6, while increasing the apoptosis marker cleaved caspase-3. Knockdown of LIMA1 using shRNA reversed the anti-tumor effects of LRP5-overexpressing osteocyte-derived CM, confirming LIMA1’s role as a key downstream effector molecule of the LRP5/Wnt pathway. Co-immunoprecipitation and immunofluorescence co-localization analyses revealed a specific interaction and spatial co-localization between exogenous LIMA1 and MYO5B in breast cancer cells, demonstrating that MYO5B is required for LIMA1-mediated inhibition of tumor cell migration and invasion. The anti-tumor effect of LRP5-overexpressing osteocyte-derived CM was attenuated upon MYO5B knockdown, and functional rescue experiments further confirmed that the integrity of the LIMA1-MYO5B axis is essential for the anti-tumor activity of LRP5-overexpressing osteocyte-derived CM. Notably, LIMA1 not only directly suppresses malignant phenotypes in tumor cells but also modulates immune cell function within the tumor microenvironment. It drives J774A.1 macrophages toward an M1 phenotype, thereby promoting an anti-tumor immune response [28]. In parallel, it inhibits osteoclastogenesis, helping to maintain the dynamic balance between bone resorption and formation and disrupting a critical step in bone metastasis [29]. In mouse models, LRP5-overexpressing osteocyte-derived CM, acting through the LIMA1–MYO5B axis, inhibited mammary tumor growth and tibial osteolysis. Micro-CT imaging demonstrated preservation of trabecular bone structure, and histological analysis showed significantly reduced tumor infiltration areas. Together, these findings suggest that the LRP5/LIMA1–MYO5B signaling axis exerts its inhibitory effects on breast cancer bone metastasis via a multi-target, synergistic mechanism, directly restraining tumor cell invasion while simultaneously remodeling the immune and bone metabolic microenvironments.

This discovery clarifies the precise molecular targets through which osteocytes act on tumor cells and establishes a transcellular regulatory network capable of disrupting the “vicious cycle” between tumor cells and the bone microenvironment [30]. Previous work has largely focused either on intrinsic tumor cell signaling or on direct pharmacological inhibition of osteoclasts. In contrast, our study uniquely targets tumor cells indirectly by modulating osteocytic LIMA1 secretion and its downstream interaction with MYO5B in cancer cells. By positioning LRP5/LIMA1/MYO5B as a functional axis linking osteocytes to tumor cells, we provide a mechanistic framework in which osteocytes are not passive bystanders but active organizers of local anti-tumor responses in bone. Furthermore, although MYO5B has been implicated in vesicle trafficking and epithelial polarity in other tissues, its role in breast cancer biology has remained largely unexplored. Our work is, to our knowledge, the first to systematically investigate MYO5B as a critical mediator of osteocyte-derived signals in breast cancer, thereby expanding the functional repertoire of both MYO5B and osteocytic Wnt signaling in the context of bone metastasis.

To our knowledge, this is the first study identifying the LRP5-LIMA1-MYO5B signaling axis as a potential therapeutic target for breast cancer bone metastasis [31,32]. Rather than exclusively inhibiting tumor cell-intrinsic pathways, our strategy directly modulates the bone microenvironment through osteocytic LRP5 overexpression, thereby reprogramming the “soil” in which metastatic cells reside. LIMA1 emerges as a dual-function effector downstream of LRP5: it exerts cell-autonomous effects by directly constraining tumor cell proliferation, migration, and invasion, and non-cell-autonomous effects by modulating osteoclast differentiation, endothelial tube formation, and macrophage polarization [33]. Our experimental findings hint at the possibility of a novel osteocyte-based cancer treatment strategy [34]. Through these combined actions, LRP5-activated osteocytes, via LIMA1 and MYO5B, simultaneously suppress tumor growth, limit osteolysis, and alleviate the immunosuppressive milieu within the bone metastatic niche [35,36]. These findings support the concept that targeting osteocyte-centered signaling may offer multi-dimensional control over tumor cells, bone remodeling, and anti-tumor immunity.

This study has several limitations. The pathway through which LIMA1 is secreted extracellularly is not yet fully elucidated. Previous studies suggest that LIMA1 can be secreted via extracellular vesicles [37,38]. We hypothesize that LIMA1 secreted by osteocytes may exert its effects by binding to MYO5B in tumor cells through mechanisms such as membrane fusion or endocytosis. Therefore, our future research will focus on elucidating the specific secretory pathway of LIMA1 and the precise molecular mechanism underlying its interaction with MYO5B. Murine cell lines and mouse models were selected to delineate a potential osteocyte-centered mechanism with translational relevance to human breast cancer bone metastasis. However, several limitations should be acknowledged. First, although EO771 cells and C57BL/6 mice provide an immunocompetent, syngeneic setting, interspecies differences in osteocyte biology, immune composition, and bone remodeling may limit direct extrapolation to patients [39,40]. Second, many of our in vitro experiments rely on two-dimensional culture conditions and conditioned medium, which do not fully recapitulate the three-dimensional architecture and mechanical cues of the bone metastatic niche, nor the complex, spatially organized interactions among osteoblasts/osteocytes, osteoclasts, mesenchymal stem cells, endothelial cells, and diverse immune cell subsets. As a result, the magnitude and integration of LRP5–LIMA1–MYO5B–mediated effects in humans may be underestimated or qualitatively different. Future studies should therefore validate this mechanism in primary human osteocytes and breast cancer specimens, and ideally in more physiologically relevant systems such as humanized mouse models, organotypic bone cultures, or microfluidic bone-on-a-chip platforms, combined with multi-omics and spatial profiling approaches, to rigorously assess its clinical translational relevance [41,42].

This study demonstrates that osteocyte-derived conditioned medium, when LRP5 is overexpressed, not only directly restrains tumor cell aggressiveness but also reprograms the “soil” of the bone microenvironment, thereby achieving multi-faceted therapeutic benefits. The core clinical implication of targeting the LRP5/LIMA1/MYO5B axis lies in its dual efficacy: simultaneously inhibiting metastatic tumor growth and repairing tumor-induced bone destruction, ultimately restoring a healthier bone microenvironment—an outcome that is difficult to achieve with single-agent cytotoxic or anti-resorptive therapies [43]. Because this strategy acts primarily by modulating host stromal and immune components rather than imposing strong direct selective pressure on tumor cells, it may also reduce the risk of acquired drug resistance and provide more durable responses. In addition, focusing on osteocyte-centered signaling opens opportunities for high-precision, low-toxicity interventions. For example, bone-targeted nanocarriers or biomaterials could be engineered to locally enhance LRP5 signaling, deliver recombinant LIMA1, or modulate MYO5B-dependent pathways specifically at sites of bone metastasis, thereby maximizing on-target efficacy while minimizing systemic toxicity [44,45]. Taken together, these findings not only propose a new conceptual strategy for treating breast cancer bone metastasis by “healing the soil” rather than solely “killing the seeds” but also provide a mechanistic platform for the future development of osteocyte-based or bone-targeted combination therapies aimed at remodeling the bone metastatic microenvironment.

## 4. Materials and Methods

### 4.1. Cell Culture

EO771 mouse mammary carcinoma cells, RAW264.7 pre-preosteoclast cells, HUVEC human umbilical vein endothelial cells (from Procell Life Science & Technology, Wuhan, China), and J774A.1 murine monocytic macrophages (from FUHENG BIOLOGY, Shanghai, China) were cultured in vitro using were cultured in high-glucose Dulbecco’s modified Eagle medium (DMEM; Servicebio, G4515, Wuhan, China) supplemented with 10% fetal bovine serum (FBS; Sigma, F8687, Saint Louis, MO, USA) and 1% penicillin–streptomycin (100 U/mL penicillin and 100 μg/mL streptomycin). MLO-A5 osteoblast cells (a generous gift from Prof. L. Bonewald, Indiana University, Indianapolis, IN, USA) were cultured in α-minimum essential medium (α-MEM; Servicebio, G4554, Wuhan, China) containing 10% FBS and 1% penicillin–streptomycin. All cell lines were incubated at 37 °C in a humidified atmosphere with 5% CO_2_.

### 4.2. Transfection and Preparation of CM

LRP5/MYO5B overexpression and LIMA1/MYO5B knockdown models (Custom-made by OBiO Technology, Shanghai, China) were established in MLO-A5 and EO771 cells. Cells were transfected with expression plasmids or empty vector controls using Lipofectamine™ 3000 (Thermo Fisher Scientific, L3000015, Waltham, MA, USA) or infected with lentiviral short hairpin RNAs (shRNAs) targeting LIMA1 or MYO5B (non-targeting shRNA as control), followed by puromycin selection to generate stable cell lines. Transfection and knockdown efficiencies were assessed 24 h after transfection by Western blotting using the following primary antibodies: LRP5 (1:1000, Cell Signaling, 5731S, Danvers, MA, USA), LIMA1 (1:5000, Proteintech, 16639-1-AP, Wuhan, China), and MYO5B (1:1000, Invitrogen, PA5-97115, Carlsbad, CA, USA). β-actin (1:5000, Sigma, A5441, USA) served as the loading control.

For preparation of conditioned medium, osteocytes were cultured to approximately 80% confluence, washed twice with PBS, and incubated in serum-free medium for 24–48 h. The supernatant was collected, centrifuged sequentially at 2000 rpm and 4000 rpm to remove cell debris, passed through a 0.22-µm filter, aliquoted, and stored at −80 °C until use.

### 4.3. Cell Viability Assay

Cells were cultured in 96-well plates at 5000 cells per well. After adhesion, they were treated with conditioned medium (CM) or recombinant protein for 24 h. Cell viability was assessed using either the CCK-8 assay (incubated with CCK-8 reagent at 37 °C for 1 h, absorbance measured at 450 nm, MeilunBio, MA0225, Liaoning, China) or the MTT assay (incubated with MTT for 4 h, absorbance measured at 570 nm, MeilunBio, MA0198, China). Relative cell viability was calculated as (OD treatment/OD control) × 100%.

### 4.4. Two-Dimensional Motility Assay

Cells in the logarithmic growth phase were cultured at a density of 8 × 10^5^ cells per well in 6-well plates and incubated until 100% confluent. A uniform scratch was created in the cell monolayer using a sterile plastic pipette tip. After washing three times gently with serum-free DMEM, the medium was replaced with CM or recombinant protein. The replaced culture medium is serum-free to eliminate the influence on cell proliferation. Images of the scratch wound were captured at 0 and 24 h at identical locations using an inverted microscope. The wound area was measured using ImageJ software (v1.52p), and the wound healing rate was calculated.

### 4.5. Invasion Assay

Cell invasion was assessed using Transwell chambers (8 µm pore size, Corning, 3428, New York, NY, USA) coated with Matrigel (1:10 dilution, Corning, 356234, USA). After serum starvation, 5 × 10^4^ cells in 200 µL serum-free medium were cultured in the upper chamber, with 800 µL CM in the lower chamber. Following 48 h of incubation, non-invading cells were removed with a cotton swab. Invaded cells on the lower membrane surface were fixed with 4% paraformaldehyde (Servicebio, G1101, China), stained with 0.1% crystal violet (MACKLIN, C805211, Shanghai, China), and counted from five random fields per membrane.

### 4.6. Colony-Formation Assay

The density of EO771 cells was adjusted to 500 cells/mL and cultured in six-well plates. Cm was replaced the next day, and then fresh medium was replaced every 48 h for 10 days. After the experiment was terminated, 4% paraformaldehyde was added for fixation at room temperature for 15 min, and then 0.1% crystal violet solution was used for staining in the dark for 10 min. The stained well plates were photographed and analyzed using ImageJ software.

### 4.7. Immunofluorescence

Cells were cultured on glass coverslips at 2.5 × 10^5^ cells per well. Upon adherence, cells were treated with CM for 24 h, then fixed with pre-cooled methanol, permeabilized with 0.1% Triton X-100, and blocked with 3% BSA. Subsequently, cells were incubated overnight at 4 °C with primary antibodies against MYO5B and LIMA1 (1:100 each), followed by incubation with fluorescent secondary antibodies in the dark. Nuclei were stained with DAPI (Servicebio, G1012, China), and coverslips were mounted for imaging using a confocal microscope.

### 4.8. Western Blot Analysis and ELISA Assay

CM-treated cells were lysed, and protein concentrations were quantified by BCA assay. Proteins were separated by SDS-PAGE, transferred to PVDF membranes (Merck, ISEQ00010, Darmstadt, Germany), and blocked with 5% non-fat milk. Membranes were probed overnight at 4 °C with the following primary antibodies: IL-6 (1:500, HUABIO, HA601051, Hangzhou, China), MMP9 (1:1000, Proteintech, 10375-2-AP, China), Snail (1:1000, Cell Signaling, 3879, USA), TGF-β1 (1:1000, Immunoway, YM8257, Ringwood Ave., San Jose, CA, USA), cleaved-caspase3 (1:1000, Cell Signaling, 9661S, USA), iNOS (1:5000, Immunoway, YM8628, USA), Arg-1 (1:5000, Immunoway, YM8217, USA), IL-1β (1:5000, Immunoway, YM8498, USA), and β-actin, followed by incubation with HRP-conjugated secondary antibodies. Signals were detected by ECL and quantified using a chemiluminescence imaging system. Secreted LIMA1 levels in the supernatant were measured using a commercial ELISA kit (MyBiosource, MBS289188, San Diego, CA, USA).

### 4.9. Co-Immunoprecipitation

Cells were lysed on ice in pre-chilled immunoprecipitation (IP) buffer supplemented with protease inhibitors. Cell lysates were incubated overnight at 4 °C with anti-LIMA1 antibody or control IgG together with protein A/G agarose beads (MedChemExpress, HY-K0202, Monmouth Junction, NJ, USA). The beads were then washed extensively with IP buffer, and bound proteins were eluted by boiling in SDS loading buffer, separated by SDS-PAGE, and analyzed by Western blotting using antibodies against LIMA1 and MYO5B.

### 4.10. Flow Cytometry

For apoptosis detection, EO771 cells were treated with CM for 24 h, digested, and resuspended. Cells were stained with Annexin V-FITC Apoptosis Detection Kit (Solarbio, CA1020, Beijing, China), then analyzed by flow cytometry.

For macrophage polarization, J774A.1 cells were treated similarly, stained with PE Anti-Mouse CD86 Antibody (Elabscience, E-AB-F0994D, Wuhan, China), followed by flow cytometric analysis to determine the proportion of CD86-positive M1-like macrophages.

### 4.11. Reactive Oxygen Species (ROS) Detection

Cells were seeded in 12-well plates, treated with CM for 24 h, and incubated with 10 µM DCFH-DA (Beyotime Biotechnology, S0033S, Shanghai, China) at 37 °C for 30 min. After washing, fluorescence was visualized under a microscope (488 nm excitation) and quantified with ImageJ to determine relative ROS production.

### 4.12. Tube Formation Assay

After EO771 cells were treated with CM for 24 h, the same CM was applied to HUVECs for 12 h. HUVECs were then trypsinized and seeded at 2.5 × 10^4^ cells/well into a 96-well plate pre-coated with GelNest™ Matrix (NEST Biotechnology, Wuxi 211252, China). Following 4 h of incubation, tube formation was observed under an inverted phase-contrast microscope, and angiogenesis parameters were quantified using the Angiogenesis Analyzer plugin in ImageJ.

### 4.13. Osteoclast Differentiation Assay

RAW264.7 cells were plated in 24-well plates at 30,000 cells/well and treated with CM and RANKL (50 ng/mL, ABclonal, RP02134, Wuhan, China), with medium replacement every three days. After 5–7 days, cells were fixed and stained using a TRAP staining kit (Servicebio, G1050, China). TRAP-positive multinucleated cells (≥3 nuclei) were counted as mature osteoclasts.

### 4.14. Mass Spectrometry-Based Proteomics Analysis

For proteomic analysis, conditioned media or cell lysate proteins were precipitated, reduced, alkylated, and digested with trypsin following a previously described protocol [46,47] with minor modifications. Peptides were analyzed using a Dionex UltiMate 3000 RSLC nano-UHPLC system coupled with a Q-Exactive mass spectrometer (Thermo Fisher Scientific, USA). Peptide mixtures were loaded onto a reverse-phase C18 analytical column and separated using a linear acetonitrile gradient [48]. MS/MS spectra were acquired in data-dependent acquisition mode. Raw MS data were processed with MaxQuant software [49], and proteins were identified by searching against the UniProt mouse database. Only proteins with at least one unique peptide and ≥2 MS/MS counts were considered for further quantitative and bioinformatic analyses.

### 4.15. Animal Models

All of the animal experiments were approved by the Institutional Animal Care and Use Committee of Capital Medical University, and the ethics number is AEEI-2025-163, date of approval: 25 April 2025. Before the experiment began, all the mice were acclimated for one week, and then they were randomly grouped using a random number table method. There are six animals in each group for both the breast cancer model and the breast cancer bone metastasis model. They are the normal group, the control CM group, the LRP5-overexpressing osteocyte-derived CM treatment group, and the LRP5-overexpressing osteocyte-derived CM group after knockdown of LIMA1, the MYO5B knockdown group, and the LRP5-overexpressing osteocyte-derived CM treatment group after knockdown of MYO5B. All experimental mice were housed five per cage with free access to food and water. All the animals were administered the drug and raised in the same location at the same time to eliminate confounders. For the mammary tumor model, six female C57BL/6 mice per group (~8 weeks old, supplied by Beijing Jinmuyang Company, Beijing, China) received a subcutaneous injection of EO771 cells (6.0 × 10^5^ cells in 50 µL PBS) into the mammary fat pad on Day 1. Starting from Day 2, mice received daily intraperitoneal injections of CM. Until the tumor grew to a volume of 1000 mm^3^. All animals were euthanized on Day 16, and tumor weight was measured.

For the tibial osteolytic model, six female C57BL/6 mice per group (~8 weeks old, supplied by Beijing Jinmuyang Company) received an intratibial injection of EO771 cells (6.0 × 10^5^ cells in 30 µL PBS) into the left tibia on Day 1. Starting from Day 2, mice received daily intraperitoneal injections of CM. On day 16, the experimental animals were euthanized, and tibial samples were collected for histological analysis and flow cytometry.

### 4.16. µCT Imaging and Histology

Harvested tibiae were fixed in 4% paraformaldehyde and subjected to high-resolution micro-computed tomography (micro-CT) scanning at a defined voxel size using Skyscan 1276 (Bruker-MicroCT, Germany). Three-dimensional reconstructions were generated, and trabecular bone parameters, including bone volume/tissue volume (BV/TV), bone mineral density (BMD), trabecular number (Tb.N), and trabecular separation (Tb.Sp), were quantified with the manufacturer’s software (CTan v1.17, Bruker-MicroCT). For histological analysis, tibiae were decalcified, embedded in paraffin, and sectioned. Sections were stained with hematoxylin and eosin (H&E) to assess tumor burden and bone architecture.

### 4.17. Statistical Analysis

In the cell experiment, we performed three independent experiments, and the data were expressed as the mean ± standard deviation. The number of animals in each group was determined based on efficacy analysis to achieve an 80% test efficacy. All the experimental animals were included in the statistics. The experimenter was aware of the groupings during the grouping and experimental phases, but a blinded analysis was employed in the data analysis process. Statistical significance was assessed by one-way analysis of variance (ANOVA), and the Bonferroni correction method was used for comparison between groups. The significance level was set at *p* < 0.05. The single asterisk and double asterisk in the figure indicate *p* < 0.05 and *p* < 0.01, respectively.

## 5. Conclusions

In summary, this study revealed that LRP5 overexpression in bone cells significantly inhibited breast cancer cell proliferation, migration, and invasion, and regulated osteoclast differentiation and immune response by activating the LIMA1/MYO5B signaling axis. In vivo, systemic administration of LRP5-overexpressing osteocyte-derived conditioned medium reduced mammary tumor burden and mitigated osteolytic bone destruction in tibial metastasis, whereas genetic disruption of LIMA1 in osteocytes or MYO5B in tumor cells abrogated these protective effects. As a newly defined tumor-suppressive pathway, the LRP5/LIMA1/MYO5B axis provides a mechanistic basis and potential therapeutic targets for breaking the “vicious cycle” between tumor cells and the bone microenvironment, thereby offering a promising strategy for the prevention and treatment of breast cancer bone metastasis.

## Data Availability

The original contributions presented in this study are included in the article/Supplementary Materials. Further inquiries can be directed to the corresponding authors.

## Supplementary Materials

The following supporting information can be downloaded at https://www.mdpi.com/article/10.3390/ijms27020777/s1.

## Abbreviations

ANOVA: analysis of variance; BMD: bone mineral density; BV/TV: bone volume/tissue volume; CM: conditioned medium; EMT: epithelial–mesenchymal transition; LIMA1: LIM domain and actin-binding protein 1; LRP5: Low-density lipoprotein receptor-related protein 5; MMP9: matrix metalloproteinase 9; MYO5B: Myosin Vb; RANKL: Receptor Activator of Nuclear Factor-κ B Ligand; ROS: Reactive oxygen species; Tb. N: trabecular number; Tb. Sp: trabecular separation; TGF-β1: transforming growth factor beta; t-SNE: t-Distributed stochastic neighbor embedding; TRAP: Tartrate-resistant acid phosphatase.
