# Supplementary material for: Inhibition of Breast Cancer Bone Metastasis by LRP5-Overexpressing Osteocytes via the LIMA1/MYO5B Signaling Axis
Source: Int J Mol Sci. 2026 Jan 13;27(2):777. doi: 10.3390/ijms27020777 (PMC12840822; doi:10.3390/ijms27020777)
Supplement: Supplementary file 1 [file ijms-27-00777-s001.zip › ijms-4006460-supplementary.pdf]

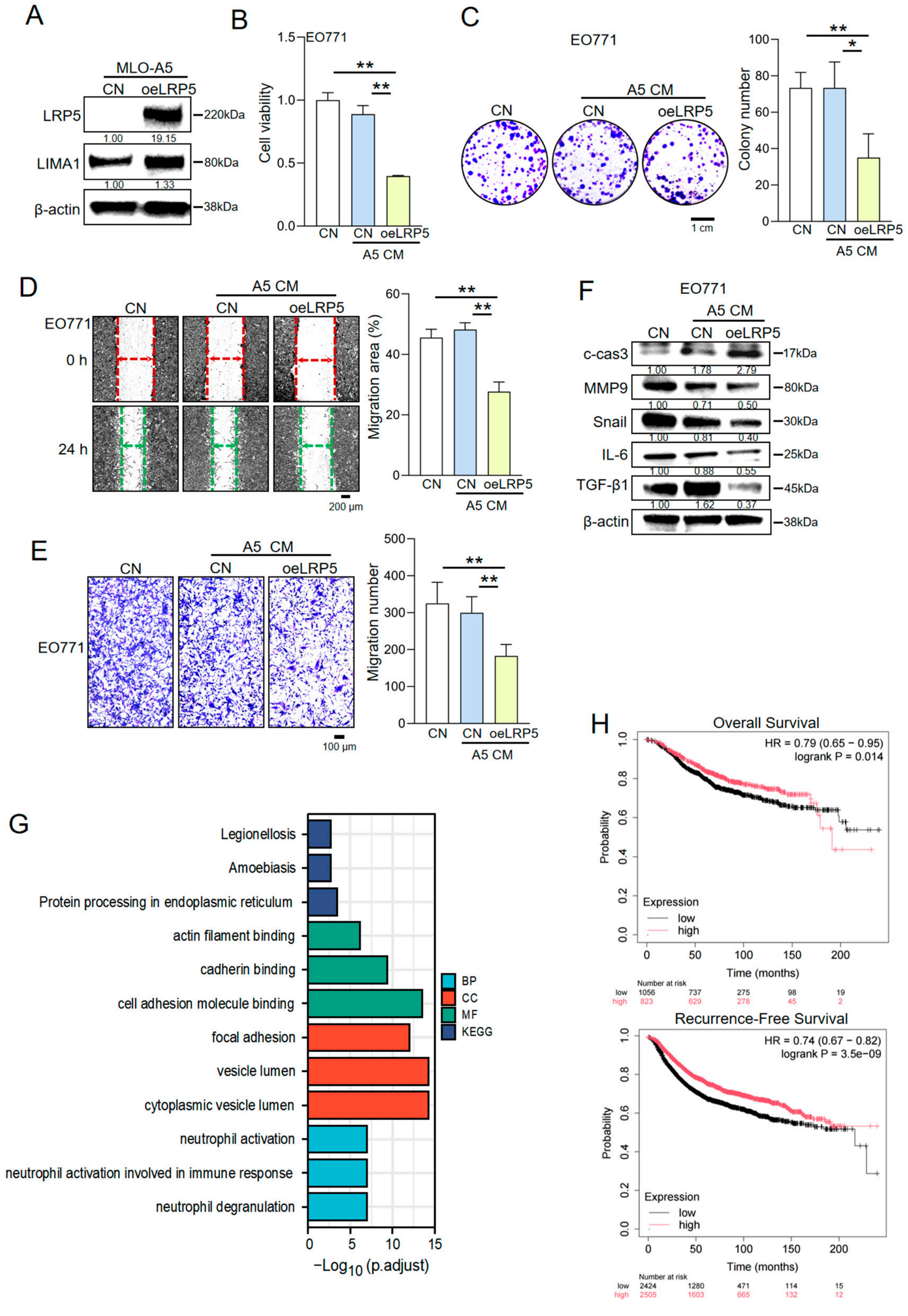

Supplementary Figure S1. Overexpression of LRP5 in MLO-A5 osteocytes enhances the tumor-suppressive capacity of their conditioned medium.

CN = Control, CM = Conditioned medium, oeLRP5 = overexpressed LRP5, BP = Biological Process, CC = Cell component, MF = Molecular function. The single and double asterisks indicate $p < 0.05$ and $p < 0.01$, respectively. (A) Plasmid-mediated LRP5 overexpression in MLO-A5 cells. (B,C) Treatment with LRP5-overexpressing osteocyte-derived CM significantly reduced EO771 cells' proliferation. (D,E) Treatment with LRP5-overexpressing osteocyte-derived CM significantly reduced EO771 cells' migration and invasion. (F) Protein levels in EO771 cells in response to LRP5-overexpressing

osteocyte-derived CM. Samples were derived from parallel experiments. (G) GO/KEGG analysis showed that secreted proteins from Lrp5-overexpressing osteocytes were enriched in cell motility and adhesion. (H) Association between LIMA1 expression and survival (OS/RFS) in breast cancer patients (Kaplan-Meier analysis).

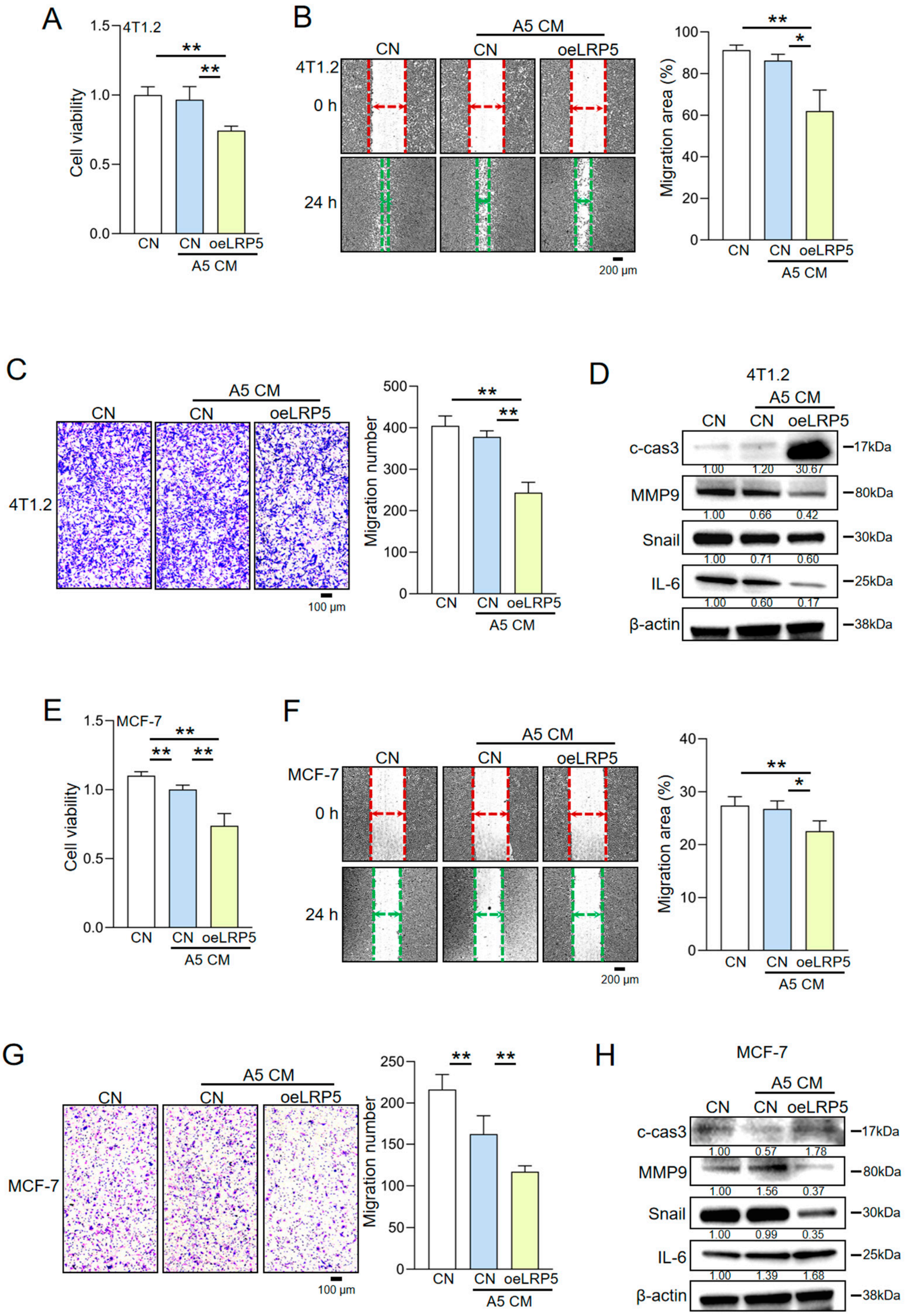

Supplementary Figure S2. Inhibitory effect of LRP5-overexpressing osteocyte-derived CM on 4T1.2 and MCF-7 cells. CN = Control, CM = Conditioned medium, oeLRP5 = overexpressed LRP5. The single and double asterisks indicate $p < 0.05$ and $p < 0.01$, respectively. (A) Inhibitory effects of LRP5-overexpressing osteocyte-derived CM on 4T1.2 cell proliferation. (B,C) Inhibitory effects of LRP5-overexpressing osteocyte-derived CM on 4T1.2 cell migration and invasion. (D) Protein levels in 4T1.2 cells in response to LRP5-overexpressing osteocyte-derived CM. (E) Inhibitory effects of LRP5-overexpressing osteocyte-derived CM on MCF-7 cell proliferation. (F,G) Inhibitory effects of LRP5-overexpressing osteocyte-derived CM on MCF-7 cell migration and invasion. (H) Protein levels in MCF-7 cells in response to LRP5-overexpressing osteocyte-derived CM. Samples were derived from parallel experiments.

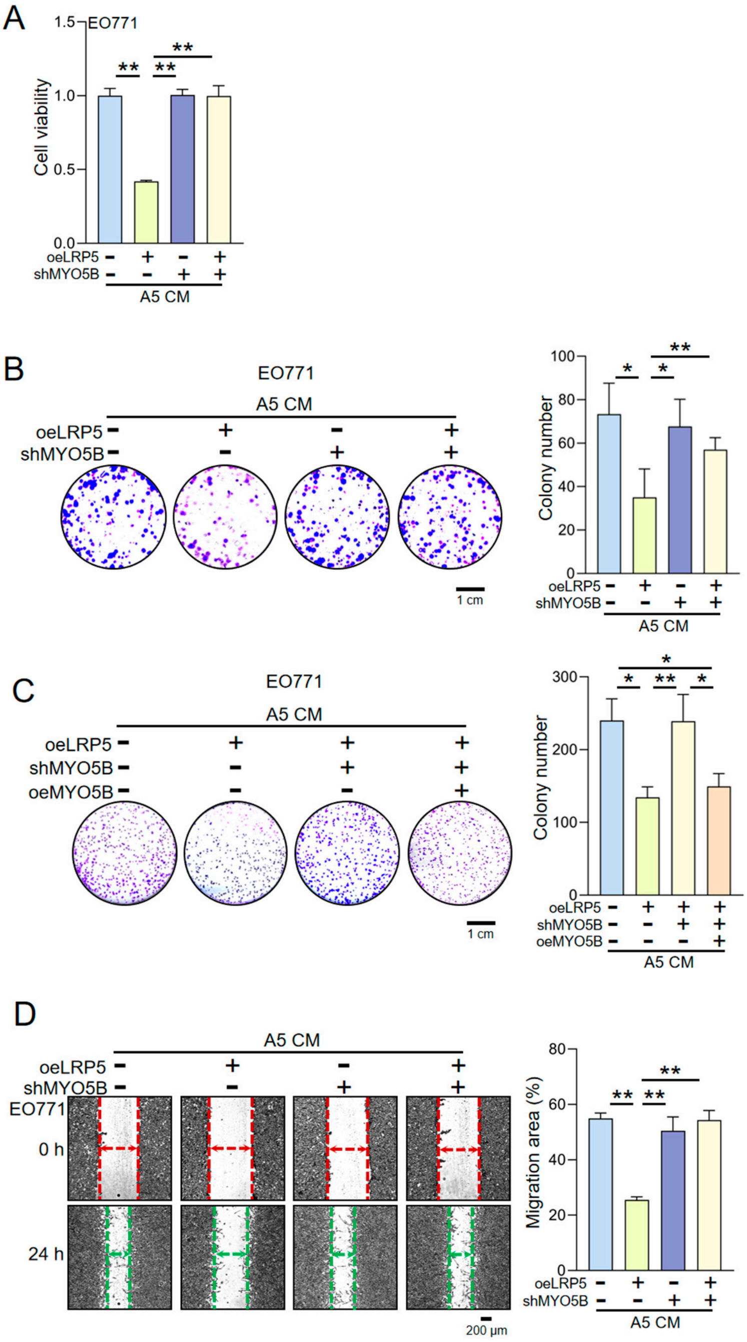

Supplementary Figure S3. Effect of LRP5-overexpressing osteocyte-derived CM on proliferation and migration of EO771 cells.

CN = Control, CM = Conditioned medium, oeLRP5 = overexpressed LRP5, shMYO5B = MYO5B shRNA, oeMYO5B = overexpressed MYO5B. The single and double asterisks indicate $p < 0.05$ and $p < 0.01$, respectively. (A) Effect of MYO5B knockdown on the proliferation of EO771 cells treated with LRP5-overexpressing osteocyte-derived CM. (B,C) Role of MYO5B in the suppression of EO771 colony formation by LRP5-overexpressing osteocyte-derived CM. (D) Role of MYO5B in the inhibition of EO771 cell migration by LRP5-overexpressing osteocyte-derived CM.

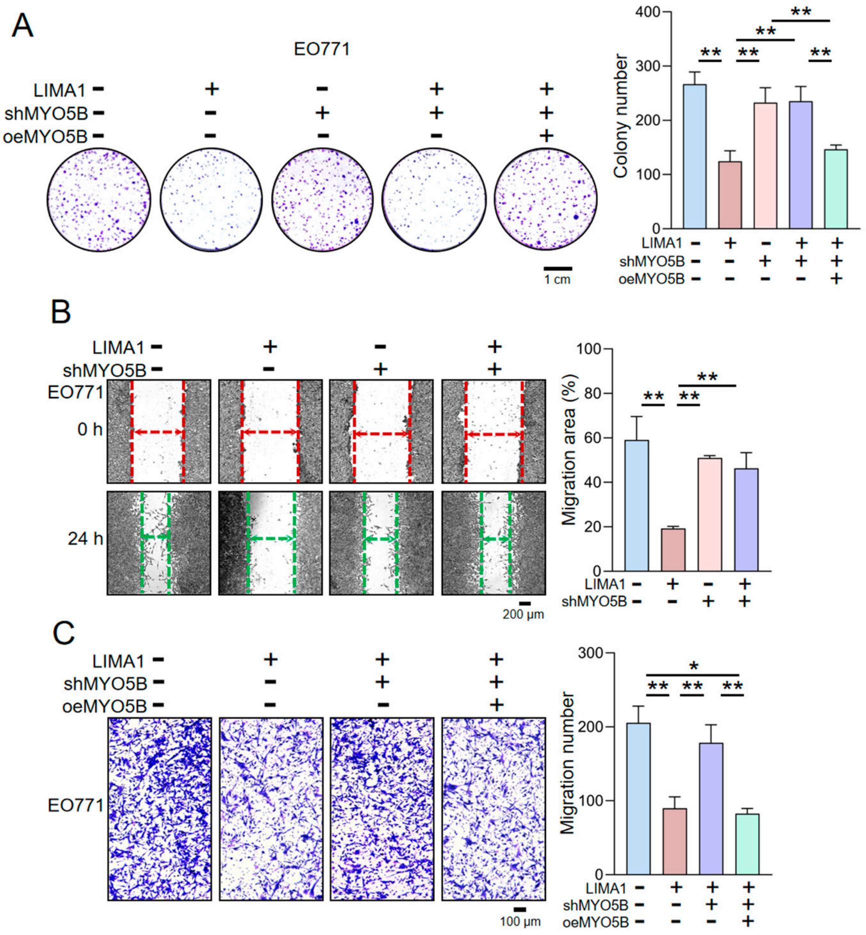

Supplementary Figure S4. Effect of MYO5B on the tumor-suppressive capacity of recombinant LIMA1 protein.

CN = Control, CM = Conditioned medium, shMYO5B = MYO5B shRNA, oeMYO5B = overexpressed MYO5B. The single and double asterisks indicate $p < 0.05$ and $p < 0.01$, respectively. (A) LIMA1 expression and proliferation of EO771 cells with MYO5B re-expression. (B,C) LIMA1 expression and migration/invasion of EO771 cells with MYO5B re-expression.

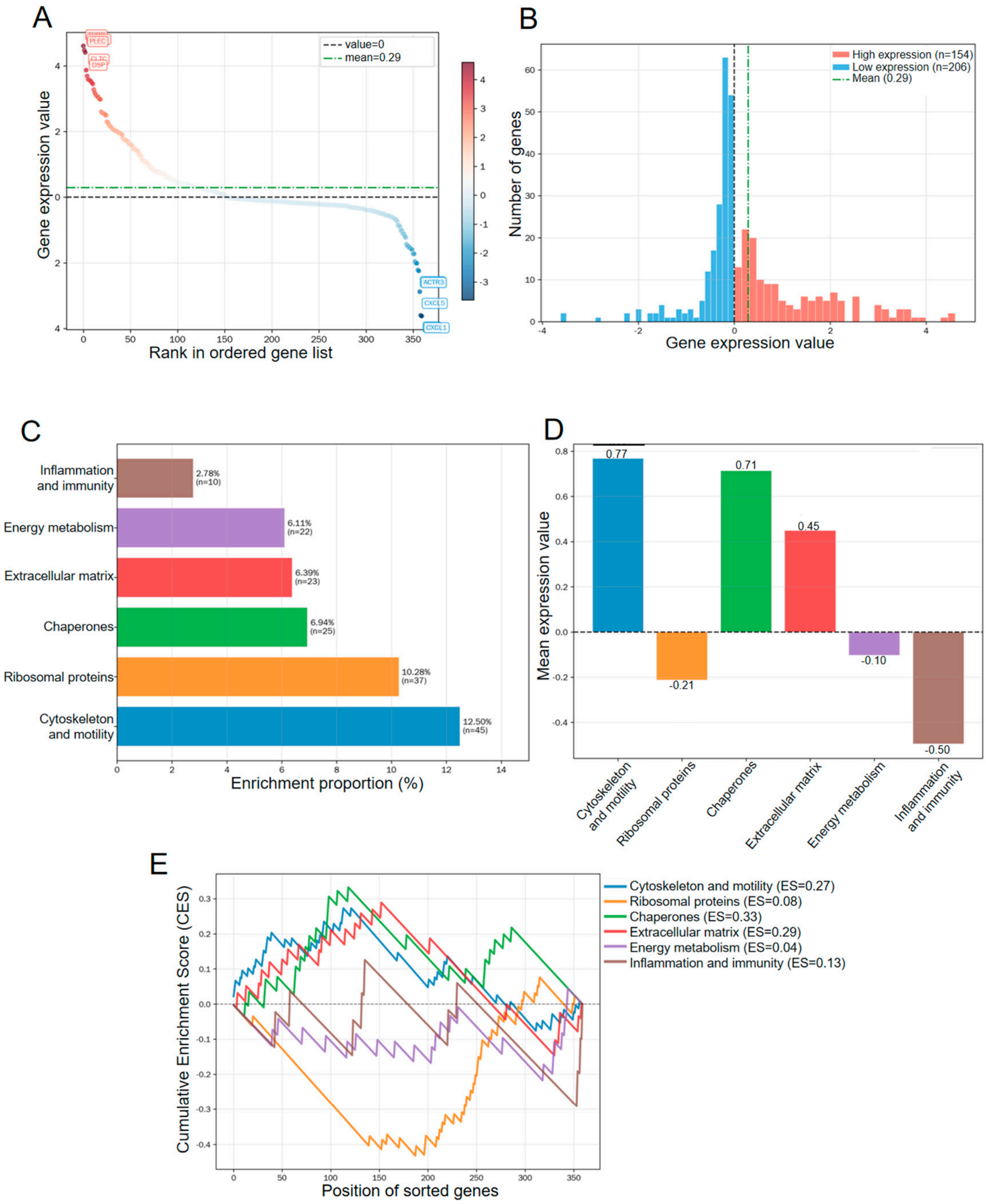

Supplementary Figure S5. Comprehensive Analysis and Visualization Report of GSEA and Functional Enrichment.

(A) Ranking of gene expression values. (B) Distribution of gene expression values. (C) Enrichment proportion of genes across functional categories. (D) Average expression values per functional category. (E) GSEA enrichment score curves for each functional category.
